# A First Look at the Research Landscape of the Neurosurgery Match in a Pass/Fail Step 1 Era: A 2024 Match Analysis

**DOI:** 10.7759/cureus.88701

**Published:** 2025-07-24

**Authors:** Eli Berglas, Jakob Liker, Aaron B Lavi, Zoe A Lainis, Rachel Berglas, Haroun Haque, Adrii Del Rosario, Eliyahu M Kochman, Matthew A Adamo

**Affiliations:** 1 Medicine, State University of New York Downstate Health Sciences University, Brooklyn, USA; 2 Medicine, Albert Einstein College of Medicine, Bronx, USA; 3 Neurosurgery, Albany Medical Center, Albany, USA

**Keywords:** education, graduate medical education, neurosurgery, nrmp match, research

## Abstract

Objective

The 2024 Residency Match was the first in recent history where most applicants did not report a numerical United States Medical Licensing Examination Step 1 score. This study will quantify the effects the scoring change had on the research productivity of successfully matched neurosurgery applicants.

Methods

Data on sex, MD/PhD status, medical school attended, and residency program were collected. Articles were categorized based on authorship, relation to neurosurgery, type of article, and the h5-index of the journal. Differences were evaluated based on sex, top 40 National Institutes of Health-funded medical school status, and acceptance at a top 30 residency program.

Results

This study evaluated the publications of 181 out of 241 matched students. They produced 2,002 articles, 85% of which were related to neurosurgery. Clinical studies were the most frequently published. The mean and median total publications were 11.1±12.8 and 8.0, respectively. On average, first-author publications accounted for 2.9±4.0 (median=2.0) of publications. Significant differences in publication metrics were found when comparing based on sex and matching into a top 30 residency program.

Conclusions

The transition of the Step 1 scoring system to Pass/Fail amplified the emphasis successful applicants placed on research. The need for an extensive research portfolio has only become greater. Results of this study may also suggest that the change to Step 1 scoring may not have lessened the burden on medical students but rather shifted it elsewhere.

## Introduction

The United States Neurosurgery Match through the National Resident Matching Program (NRMP) continues to increase in competitiveness. The 2024 match had a 68% match rate among MD seniors, down from 78% the previous year [1,2]. This trend is complicated by changes to the United States Medical Licensing Examination (USMLE) Step 1 exam. Before 2022, Step 1 was graded on a numerical scale, but has since transitioned to Pass/Fail. This decision was in part driven by the thought that a Pass/Fail exam would reduce student stress [3]. However, there are those suggesting the stress has not been reduced but rather redistributed [4]. The 2024 Match marked the first in recent history in which most students applied without a numerical Step 1 score [5]. Historically, numerical scores were used to measure an applicant's competitiveness [6-8]. With this marker gone, students must find other ways to stand out among an ever-growing applicant pool.

Research is seen as a somewhat objective metric of applicant competitiveness. The NRMP publishes data regarding self-reported research productivity. In 2022, matched neurosurgery applicants reported a mean of 25.5 abstracts, presentations, and publications, compared to 11.7 for unmatched [9]. This rose rapidly in 2024 to 37.4 and 31.8 for matched and unmatched MD seniors, respectively [5]. The validity of this data is limited as it is self-reported and likely heavily skewed [10-14]. Additionally, combining abstracts, presentations, and publications can be a misleading indication of research involvement. The resulting misconception is a strong contributing factor to increased stress among medical students applying to the Neurosurgery Match [15]. Therefore, there is a need to accurately represent the research productivity of successful neurosurgery applicants.

Previous studies have evaluated publication metrics. Most recently in the 2021 Match, the mean and median number of publications were 8.1 and 4.0, respectively [10]. Significant publication differences have also been found based on sex, MD/PhD status, and ranking of medical school [10-14]. There is now a need to perform similar analyses in lieu of the Step 1 scoring transition. By providing a clearer understanding of the research productivity from the 2023-2024 Neurosurgery Match, we provide future applicants with more accurate metrics that can be used to assess their competitiveness. We anticipate substantial increases in publication rates compared to prior years as students look for ways to enhance their applications.

## Materials and methods

We identified neurosurgery programs participating in the 2024 Match using the NRMP program directory [1]. To identify students, a review of the participating program's social media pages and websites was conducted. Programs in which no official announcement was found at the time of data collection (4/15/2024) were excluded. In total, 78 of the 116 programs that participated in the 2024 Match were included. We identified 181 students from these programs, representing 75% of all matched applicants [1]. All data used in this study were publicly accessible; thus, this study did not require institutional review board approval. The primary goal of this study was to quantify and categorize research production among successful neurosurgery applicants. A thorough evaluation of publications would provide a means to compare research involvement among students successfully matched in the 2024 Match, which was, for the most part, without Step 1 scores, to students in prior matches who applied with a Step 1 score.

Demographic data 

Medical school attended, sex, and PhD status (n=14 out of 21 reported by NRMP) were determined using publicly available information [5,16]. Medical school attended was stratified based on whether the school was among the top 40 National Institutes of Health (NIH)-funded schools in 2023 [5,13,17,18]. Data was stratified based on applicants matching into a top 30 program. This cutoff was established as this represents roughly the top quarter of programs participating in the match. This study captured 27 of the top 30 programs. Rankings of programs were determined using Doximity reputation rankings (Version 2023-2024) [11,14,19].

Publication data 

Only PubMed-indexed full-length articles published before 3/15/2024 were considered. Articles defined as "Letter to Editor" and "Comment" were excluded [20]. A multifaceted approach was used to attribute articles to students. Names were searched in Scopus®. Publications in Scopus® were cross-referenced to determine if they were also indexed in PubMed [21]. We began our search on Scopus® given that it robustly attributes articles to authors. However, Scopus® does not capture all PubMed-indexed articles, so we subsequently searched names in PubMed. To avoid misattributing articles, we used websites such as Scopus®, Google Scholar, ResearchGate, and LinkedIn [22-24]. These resources enabled the confirmation of affiliations. 

Categorization 

Publications were categorized by author rank, article type, and publication date. Publication date was divided into published before 9/27/2023 and published after 9/27/2023 but before 3/15/2024. The cutoff of 9/27/2023 represents the first day programs were able to review applications and thus was the value used in all comparative analyses. The extended cutoff until Match Day (3/15/2024) allowed for trend analysis. Five time periods were created based upon ending each year on September 27th. The first period was articles published before 9/27/2020, while the last included publications between 9/27/2023 and 3/15/2024. Publications in the last time frame were prorated to represent an annual value.

Article types were categorized as basic science, clinical, review, case report, or other. Basic science was defined as a study where the main focus used biochemical techniques or was translational. Clinical studies were defined as studies that analyzed patient data. Reviews consisted of articles that summarized previous literature. Case reports were defined as a description of a single patient. All articles that did not fall into a category were classified as "other". Articles were defined as neurosurgery-related if the focus was applicable to neurosurgery. In instances where the publication type or its relation to neurosurgery was not clear, an additional rater evaluated the article and came to a joint decision. Google Scholar's journal h5-index quantified journal quality [25]. Study data were managed using REDCap electronic data capture tools hosted at the State University of New York (SUNY) Downstate Health Sciences University [26,27]. 

Data analysis

A binomial test with an expected frequency of 50% determined if the sampled students were predominantly male or female. Using our sample's sex distribution as the expected frequency, a binomial test was performed to determine if the top 30 programs matched a greater number of female applicants. To assess how our sample represented the 2024 matched students, an additional binomial test was performed using the NRMP's reported value of 30.1% as the expected frequency of students from the top 40 NIH-funded schools [5].

Descriptive statistics, including mean (M)±standard deviation and median (Mdn), were used to describe individual-level data. Welch's t-tests were used in three comparisons. The first evaluated sex differences in total, first-author, neurosurgery-related, basic science, and clinical publications and average journal h5-index. The second and third evaluated the same publication metrics but compared students based on attendance at a top 40 NIH-funded medical school and matching at a top 30 residency program. Given that these six publication metrics overlap, Bonferroni correction was applied, and a new alpha level of 0.0083 was considered significant. To determine if the number of publications by a student affected the average h5-index of journals in which they had articles published, an R^2^ value was calculated. P-values <0.05 were considered significant except when indicated otherwise. All statistical analyses were done using IBM SPSS Statistics for Windows, Version 29.0.0.0 (IBM Corp., Armonk, New York, United States).

## Results

The 181 students captured in this study produced a total of 2,002 publications by 9/27/2023. Most of the students included in this study were male (65.8%; p<0.0001). Using 65.8% as the expected frequency, the number of male students who matched in the top 30 programs (n=58; 69%) was not significantly different from female students (n=26; 31%) matching at these programs (p=0.30). This study's sample of students consisted of significantly more students (n=77; 42.5%) from the top 40 NIH-funded medical schools when using NRMP-reported data (30.1%) to establish the expected proportion (p<0.001). 

**Figure 1:**
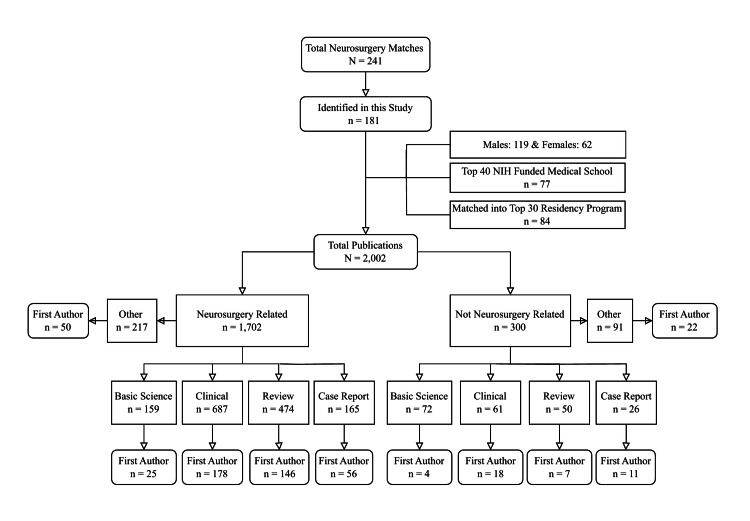
Distribution of publications by their relation to neurosurgery and article type Articles were published before 9/27/2023 by successful neurosurgery applicants in the 2024 Match. NIH: National Institutes of Health

The distribution of these articles by their relation to neurosurgery, study type, and number of first-author publications can be seen in Figure 1. The majority (85%) centered around neurosurgery-related topics. Clinical papers (n=687) comprised most of the 1,702 neurosurgery-related papers (40.4%). Additionally, clinical papers had the highest frequency of first-author neurosurgery-related publications (n=178). Despite basic science papers (n=72) having the second greatest number of articles in this category, it had the lowest proportion of first-author publications (5.6%).

**Figure 2:**
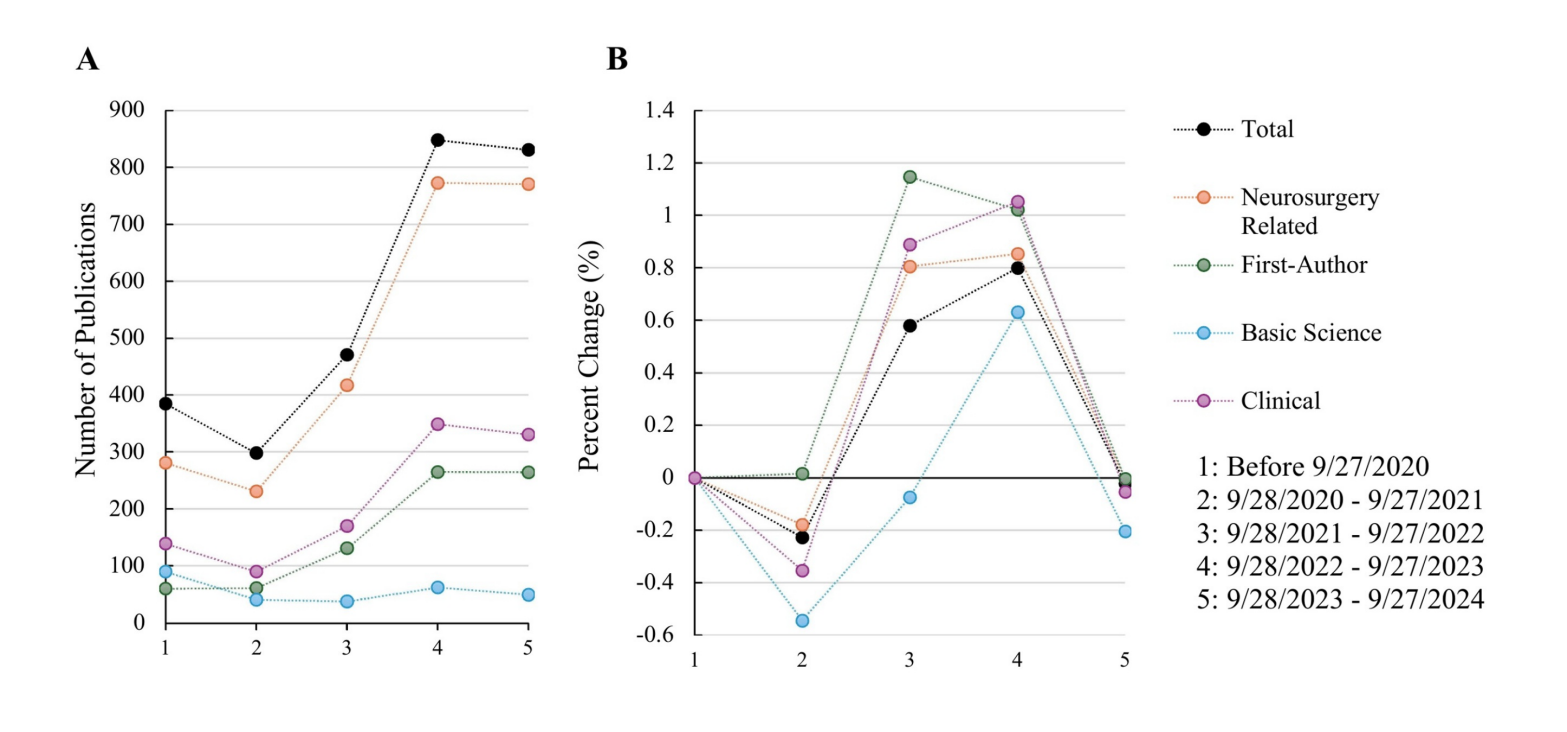
Trends of (A) publications over five time periods and (B) percent change Percent change represents a given period compared to the previous period. Period 5 was prorated using publications per day (until 3/15/2024) to project a year's worth of publications.

Figure 2 demonstrates the trends of total, neurosurgery-related, first-author, basic science, and clinical publications over five time frames. The trends of total publications over time closely matched those of neurosurgery-related publications. Similarly, first-author and clinical publications demonstrated similar counts over time (Figure 2A). Basic science publications showed slower growth; this was most apparent between 9/28/2020 through 9/27/2022 as represented by the percent change in period 3 (Figure 2B).

The percentile breakdown of publication metrics is shown in Table 1. The mean number of publications was 11.1±12.8 (Mdn=8.0), of which 2.9±4.0 (Mdn=2.0) were first-author. Eleven students had no recorded publications. The 14 students who held an MD/PhD contributed above-average metrics in all categories, except for clinical publications. MD/PhD students had an average of 13.5±12.3 (Mdn=9.0) total publications, 3.5±3.6 (Mdn=3.0) as first author, 8.5±8.9 (Mdn=5.5) in the top three authors, 12.2±11.8 (Mdn=6.5) related to neurosurgery, 3.1±2.8 (Mdn=3.0) in basic science, and 3.7±5.9 (Mdn=1.0) in clinical publications. Additionally, the publications of MD/PhD students were in journals with an average h5-index of 94.8±26.4 (Mdn=93.9).

**Table 1:** Percentile rankings of seven publication metrics for 181 students successful in the 2024 Neurosurgery Match x̅: mean±standard deviation; NS: neurosurgery

Percentiles	Total x̅=11.1±12.8	First author x̅=2.9±4.0	Top three author x̅=7.0±8.8	NS-related x̅=9.4±11.7	Basic science x̅=1.3±2.1	Clinical x̅=4.1±7.2	Average journal h5-index x̅=61.2±30.9
90th	28	7	16	24	4	11	105
80th	16	4	11	14	2	6	82
70th	12	3	8	10	1	4	66
60th	10	3	6	8	1	2	59
50th (median)	8	2	4	6	0	1	56
40th	5	1	3	4	0	1	52
30th	4	0	2	3	0	0	49
20th	2	0	1	1	0	0	44
10th	1	0	0	1	0	0	33

**Figure 3:**
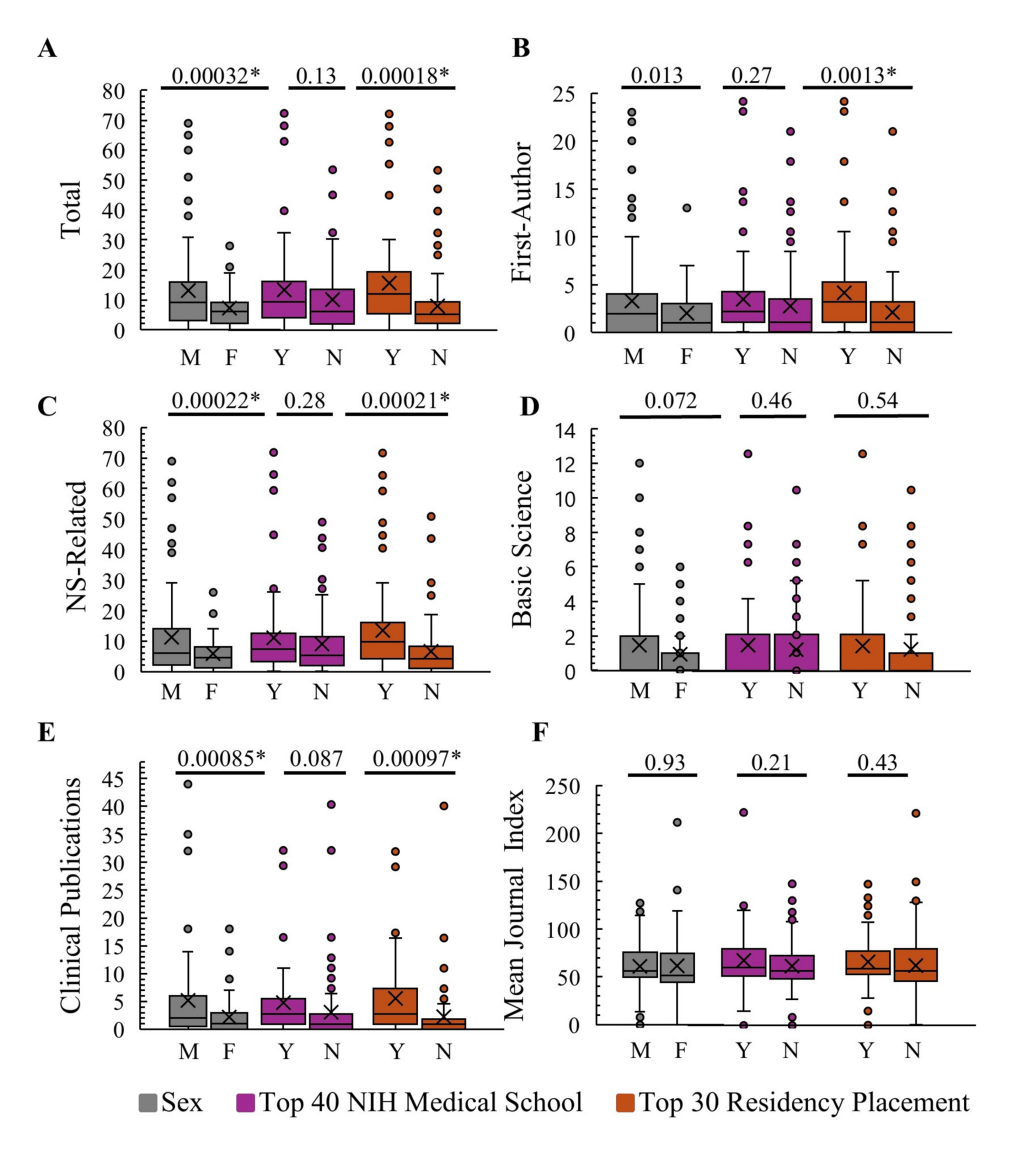
Publication distributions for comparisons by (A) total, (B) first-author, (C) neurosurgery-related, (D) basic science, and (E) clinical publications and (F) average journal h5-index Comparisons were made based on (1) male or female, (2) graduation from a medical school with top 40 NIH funding in 2023, and (3) matching at a top 30 residency program as ranked by Doximity. The values above the line denote p-value for Welch's t-tests. The asterisk indicates significance compared to Bonferroni correction (α=0.0083). x within the figure: mean; NS: neurosurgery; M: male; F: female; Y: yes; N: no; NIH: National Institutes of Health

**Figure 4:**
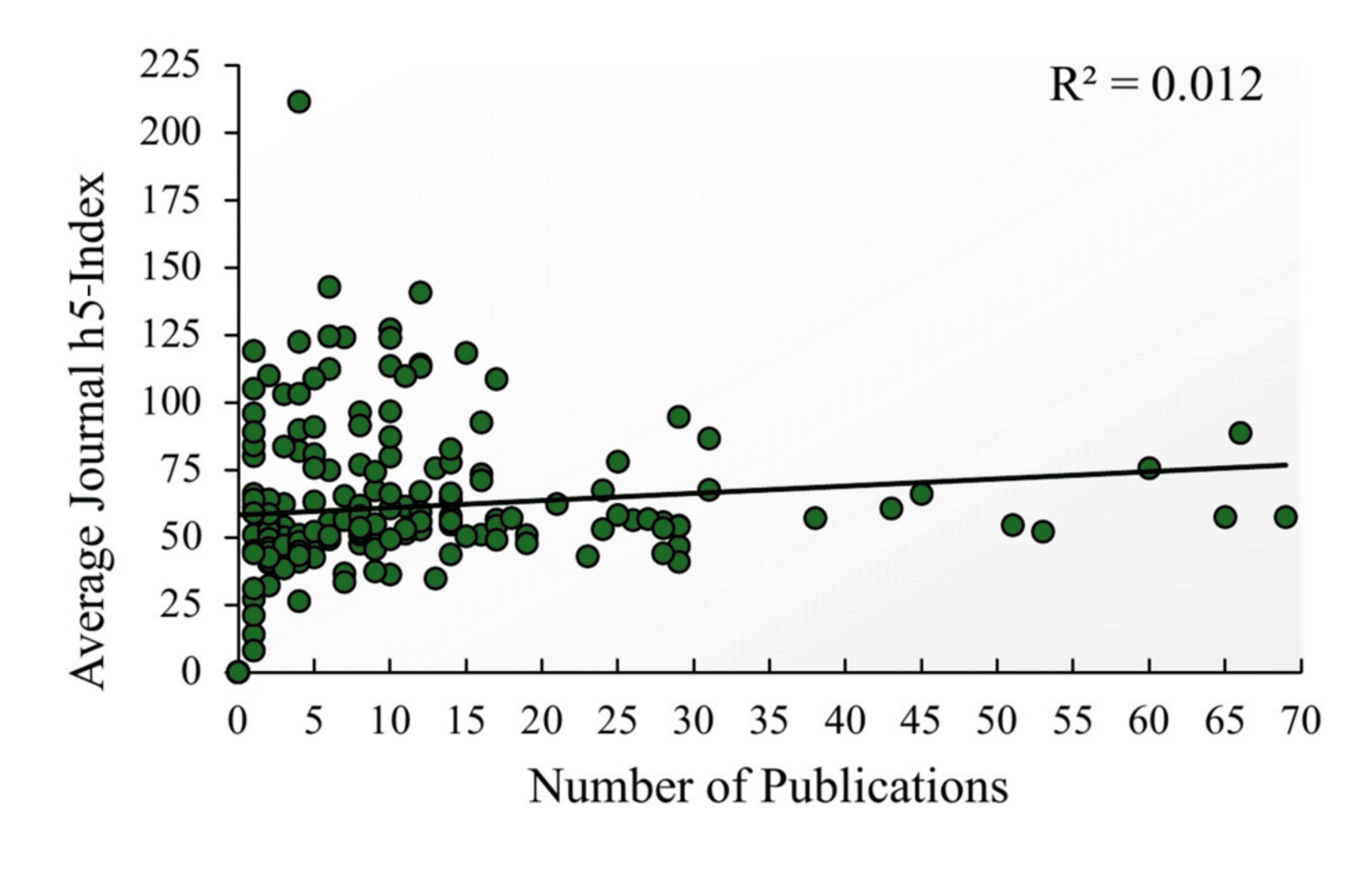
Correlation of number of publications by matched neurosurgery applicants with average journal h5-index

Figure 3A-3F displays the results of comparisons based on sex, graduation from top 40 NIH-funded medical schools, and matching into a top 30 residency. These tests considered α=0.0083 as the significance threshold following adjustment for multiple testing. Significant comparisons are summarized as follows: Male students had greater total (p=0.00032), neurosurgery-related (p=0.00022), and clinical publications (p=0.00085) than female students. Comparisons based on whether the student graduated from a top 40 NIH-funded medical school showed no significant results. Contrarily, students who matched into a top 30 residency program had significantly greater total (p=0.00018), first-author (p=0.0013), neurosurgery-related (p=0.00021), and clinical publications (p=0.00097). Lastly, there was no appreciable correlation (R2=0.012) found between an applicant's total publications and the average h5-index of the journals in which they were published (Figure 4).

## Discussion

The present study provides a first look into how the Step 1 scoring transition impacted the research production of neurosurgery applicants. Currently, only research production data provided by the NRMP is available for the 2024 Match [5]. These reported statistics are subject to bias. Given the uncertainty around how residency programs would evaluate applicants without Step 1 scores, this study provides benchmarks for future applicants to assess their competitiveness. 

This study, much like previous analyses, found publication metrics to be positively skewed with a mean of 11.1 and a median of eight publications [10-14]. Comparing these findings to Hasley et al.'s 2021 Match analysis is difficult, given that they removed students with MD/PhD dual degrees and discounted students with greater than 30 publications [10]. Therefore, it is more appropriate to compare the median, which Hasley et al. found to be four publications [10]. Despite the differences of the present study, a 100% increase in median publications is a substantial increase. To put this three-year increase into context, a study by Duy et al. found an identical median of four publications in 2019 [11]. Additionally, compared to 2021, first-author publications increased from an average of 1.4 to 2.9 in the present study [10]. These findings suggest that applicants are increasing both the quantity and their level of involvement in research. 

Unlike total and first-author publications, basic science articles have not seen a similar increase. Compared to 2018, where the mean was 1.7, the present study reported 1.28 basic science publications [13]. The MD/PhD students included in this analysis averaged 3.1 basic science publications. This value was lower than the 5.2 reported for MD/PhD students from 2011 through 2018 [13]. A possible explanation is that the increased time commitment of basic science research deters students from pursuing it. Additionally, it appears that students participating in basic science research may contribute less. This is supported by Figure 1, which shows basic science publications to have the lowest proportion of first authorship. However, as can be seen in Figure 4, the number of publications does not correlate (positively or negatively) with the average journal h5-index in which students are publishing. This provides evidence against the idea that an applicant who produces many publications inevitably sacrifices the quality of their work. Overall, these findings demonstrate the need to evaluate a student's published work based on the study type. This would provide a more contextual view of their respective research output. A more holistic view of study types would also provide reassurance to students who want to pursue basic science research but fear fewer publications will impact their ability to match.

Our study found that 85% of all published studies were related to a neurosurgical topic. These findings are nearly identical to previous reports analyzing research output in the 2019 and 2021 Matches [10,11]. By evaluating the trend lines seen in Figure 2A, it is apparent that many students were already involved in neurosurgery-related research even before beginning medical school. While it is likely that all publications before 9/27/2020 do not indicate work done prior to medical school, this close relationship indicates a long-standing interest in neurosurgery-related topics among successful neurosurgery matriculants. 

Nearly 31% of included students were female, which is slightly greater than values from previous years [28]. We also noted that the top 30 programs matched female students similarly to the included non-top 30 programs. Neurosurgery, which has long been a male-dominated field, has begun to close the gap as the percentage of female neurosurgery residents has risen from 15.9% to 29.8% from 2016 to 2022 [29]. There are substantiated concerns that this disparity will remain without concerted efforts to enhance both the mentorship and recruitment of female students [28,29]. In the present study, female students had significantly fewer total, neurosurgery-related, and clinical publications. There is a clear difference in publication totals among female applicants compared to the 2021 data. Hasley et al. specified that females had a median of 2.5 publications compared to 6.0 found in the present study [10]. These values indicate that all applicants, regardless of sex, are striving to enhance their research portfolios. 

The same findings are true for students in lower NIH-funded medical schools. Previous studies have noted significant publication differences based on NIH funding ranking; this was not the case in the present study (Figure 3) [13,14]. This can be attributed to the growing awareness that students from lower-ranked schools have greater difficulty in successfully matching into neurosurgery [1]. Having now lost the ability to use Step 1 as a way to separate themselves, students from lesser-funded medical schools may have prioritized increasing the quantity of their research in hopes of securing a residency spot. However, consistent with prior studies, students matching into a top 30 residency program produced significantly more publications [11,13]. This is expected given that top residency programs are within large academic hospitals, which likely place a higher value on research.

The results of the present study depict a clear picture of increased research production among matched neurosurgery applicants. However, there are limitations. The retrospective nature of this study limits the ability to determine the causality of the Step 1 exam scoring change on research production. Another limitation is that this study included a greater proportion of students who graduated from the top 40 NIH-funded medical schools. This may provide hesitancy in interpreting the sample data to be representative of all successful applicants. However, as seen in Figure 3, there were no significant differences in any included publication metric based on this category. With the sampling discrepancies not representing increases in publication metrics and previous studies having used similarly sized samples, the results are unlikely to be skewed due to this factor [10,12]. It is important to note that our sample captured a greater proportion of the top 30 programs, which did show significant publication disparities. Considering these factors, it is likely that averages of the entire sample may be inflated compared to true population values. 

The present study identified an unprecedented increase in research production among successful neurosurgery applicants. The increase in research production applied to male and female applicants. Additionally, research production rose for all students regardless of the medical school at which they graduated or the residency program in which they matched. The overall increase in research involvement coincided with the absence of a numerical Step 1 score. Thus, given that applicants were aware of the planned change to the Step 1 score several years in advance, it signifies that applicants quickly turned to increasing their research involvement as a way to increase their competitiveness. Future research should focus on continued reporting of publication metrics, as this recent rise in publications is unlikely to cease. Given that more applicants use research to gain a competitive edge over the increasing number of applicants, greater consideration of the Step 1 scoring change may be necessary. As students continue to adapt, their stress levels are likely to rise as they continually push their boundaries in hopes of securing a residency spot. We also encourage the NRMP to enhance its reporting of Match statistics. Likewise, residency programs can also improve their transparency by clarifying the attributes they look for in applicants. 

## Conclusions

Total and first-author publications experienced a dramatic increase in the 2024 Match. This was true of all successful students regardless of sex or school ranking. Despite a relative movement away from basic science research, the prestige of journals in which these students are publishing remains strong. Research requirements needed to match into the top residency programs remain higher; however, the overall shift in the research landscape of the neurosurgery match has impacted all residency programs. The findings of this study call into question whether ridding Step 1 of a numerical score has truly reduced the burden on medical students or simply shifted it elsewhere.
